# Formulation and characterization of glipizide solid dosage form with enhanced solubility

**DOI:** 10.1371/journal.pone.0297467

**Published:** 2024-02-23

**Authors:** Badriyah Shadid Alotaibi, Muhammad Ahsan Khan, Kaleem Ullah, Haya Yasin, Abdul Mannan, Shujaat Ali Khan, Ghulam Murtaza

**Affiliations:** 1 Department of Pharmaceutical Sciences, College of Pharmacy, Princess Nourah Bint Abdulrahman University, Riyadh, Saudi Arabia; 2 Department of Pharmacy, COMSATS University Islamabad, Abbottabad Campus, Abbottabad, Pakistan; 3 Faculty of Pharmacy, Hamdard University, Islamabad Campus, Pakistan; 4 Department of Pharmaceutical Sciences, College of Pharmacy and Health Sciences, Ajman University, Ajman, United Arab Emirates; 5 Department of Pharmacy, COMSATS University Islamabad, Lahore Campus, Lahore, Pakistan; Egaz Moniz School of Health and Science, PORTUGAL

## Abstract

Glipizide, a poor water-soluble drug belongs to BCS class II. The proposed work aimed to enhance the solubility of glipizide by preparing solid dispersions, using polyvinyl pyrrolidone (PVP) and polyethylene glycol (PEG). Solvent evaporation method was used for the preparation of glipizide solid dispersions. Solid dispersions were prepared in four different drug-to-polymer ratios i.e. 1:1, 1:2, 1:3 and 1:4. Mainly effect of three polymers (PVP K30, PVP K90 and PEG 6000) was evaluated on the solubility and dissolution of glipizide. The *in-vitro* dissolution of all prepared formulations was performed under pH 6.8 at 37°C using USP type II apparatus. *In-vitro* dissolution results revealed that the formulations having high concentrations of the polymer showed enhanced solubility. Enhancements in the solubility and rate of dissolution of the drug were noted in solid dispersion formulations compared to the physical blends and pure drug. Solid dispersions containing polyvinyl pyrrolidone exhibited a more favorable pattern of drug release compared to the corresponding solid dispersions with PEG. An increase in the maximum solubility of the drug within the solid dispersion systems was observed in all instances. Two solid dispersion formulations were optimized and formulated into immediate-release tablets, which passed all the pharmacopoeial and non-pharmacopoeial tests. Fourier transformed Infrared (FTIR) spectroscopy X-ray diffraction (XRD) and Differential scanning calorimetry (DSC) were used to indicate drug: polymer interactions in solid state. Analysis of the solid dispersion samples through characterization tests indicated the compatibility between the drug and the polymer.

## 1. Introduction

Following oral administration, the active drug dissolves initially in the gastric or intestinal fluids. Subsequently, it traverses the membranes of the gastrointestinal tract (GIT) to enter the systemic circulation. As a result, the extent to which the drug is bioavailable hinges on its solubility and rate of dissolution, with solubility acting as the constraining factor. In cases where a drug exhibits an exceptionally sluggish dissolution rate, requiring more time to traverse its absorptive sites than the transit time, it falls into the category of poorly soluble compounds. This circumstance leads to inadequate bioavailability [1]. Around 40% of recently discovered medicinal compounds demonstrate restricted solubility in aqueous environments, leading to suboptimal and frequently inconsistent oral bioavailability [2, 3]. Hence, improving the oral bioavailability of a drug includes enhancement of drug solubility and dissolution rate, and this is the key part of pharmaceutical research [4].

Several methodologies have been employed to improve the solubility and dissolution rate of drugs with low aqueous solubility. These approaches include methods such as size reduction, co-crystal formation, polymorphic alterations, salt formation, and the utilization of amorphous systems. Among them, the solid dispersion technique is most frequently used [5]. Sekiguchi and Obi introduced the concept of solid dispersion in 1961 as a method to decrease the drug’s particle size and crystallinity, thereby enhancing the dissolution rate and absorption kinetics [6].

In accordance with Chiou and Riegelman, a solid dispersion is described as the dispersion of one or more active substances within a non-reactive carrier or matrix in a solid state. This preparation is achieved through techniques such as melting (fusion), employing a solvent, or utilizing a solvent-melting approach [7]. When dealing with solid dispersion, hydrophilic polymers such as polyvinylpyrrolidone (PVP), polyethylene glycol (PEG), and hydroxypropyl-methylcellulose (HPMC) have often played a significant role as carriers [8, 9].

Glipizide (C_21_H_27_N_5_O_4_S) is an anti-diabetic drug belongs to sulphonyl urea and commonly used to lower blood glucose level in diabetes mellitus type II patients. It is very slightly soluble in methylene chloride, practically insoluble in water and absolute ethanol. It dissolves in dilute solutions of alkali hydroxides. Glipizide belongs to BCS II, its half-life is 2‐4 hrs. Poor soluble drugs like glipizide shows variable bioavailability as their limiting factor is dissolution rate [9]. Previous studies have reported glipizide solubility enhancement by inclusion complex formation with β-cyclodextrin [10] and surface solid dispersion by using Kollidon CL [11].

In this study we have used polyethylene glycol (PEG 6000) and two grades of polyvinylpyrrolidone (i.e. PVP K30 and PVP K90). Core characteristics of PVP (polyvinylpyrrolidone) involve its creation through polymerization, resulting in molecular weights (MW) ranging from 2500 to 3,000,000. The length of the PVP chain plays a vital role in determining how quickly the drug dissolves within the solid dispersion. As the chain length increases, the ability of PVPs to dissolve in water decreases [12]. Sharma and Jain utilized the solvent method to create a solid dispersion of carvedilol with PVP K30, leading to a remarkable 35-fold improvement in the solubility of carvedilol [13]. In a separate investigation, Tantishaiyakul successfully formulated solid dispersions of the poorly water-soluble drug piroxicam using PVP K30. This approach resulted in an impressive 38-fold enhancement in the drug’s dissolution rate [14]. A recent study explored Poly(2-ethyl-2-oxazoline) (PEOX) as an alternative to PVP for forming solid dispersions with the poorly soluble drug glipizide. PEOX demonstrated a strong inhibitory effect on crystal nucleation kinetics, resulting in significantly improved solubility and dissolution rates, particularly with PEOX-5K, suggesting its potential for enhancing the oral bioavailability of poorly soluble drugs [15].

In contrast, polyethylene glycols are polymers originating from ethylene oxide, exhibiting molecular weights spanning a spectrum from 200 to 300,000. When formulating solutions and solid dispersions, PEGs within the MW range of 1500 to 20,000 are frequently chosen. As MW increases, the polymer’s viscosity also rises. PEG usually exhibits good water solubility; however, this solubility diminishes as MW increases. PEGs with MW values between 4000 and 6000 are commonly selected for generating solid dispersions, as they maintain a notably high level of water solubility within this range [9]. Utilization of PEG with lower molecular weight results in the formation of a sticky product, posing challenges in its transformation into a pharmaceutical dosage form. Conversely, the application of PEGs with higher molecular weights has demonstrated successful outcomes in addressing this issue [16]. Patel et al. conducted a study focused on improving the dissolution rate of the poorly water-soluble drug glipizide by utilizing solid dispersion with PEG 6000, PEG 8000, and PXM 188 as carriers, ultimately demonstrating enhanced blood glucose reduction in preclinical testing, particularly with the use of PXM 188 [17].

The primary aim of this study was to explore the potential for enhancing the solubility and dissolution rate of glipizide solid dosage form. This was achieved by creating solid dispersions through the utilization of PVP K30, PVP K90, and PEG 6000 via the solvent evaporation method. To comprehensively characterize the resulting dispersions, an analysis was conducted using techniques such as FTIR, XRD, and DSC. Additionally, dissolution and solubility studies were performed to further evaluate the formulations.

## 2. Methodology

### Materials

Glipizide (99.9%) was gifted by Pharmedic Pharmaceuticals, Karachi, Pakistan. PVP K90 were gifted by Global Pharmaceuticals, Islamabad, Pakistan. PVP K30 and PEG 6000 was gifted by National Agriculture Research Center, Islamabad, Pakistan. Methanol, ethanol and dichloromethane was obtained from Daejung, Korea. Distilled water was prepared inhouse at Pharmaceutics Lab, Department of Pharmacy, COMSATS University Islamabad, Abbottabad Campus, Pakistan.

### Methods

#### 2.1 Preparation of physical mixture

Glipizide and polymers (PVP K30, K90, and PEG 6000), individually sieved through a 100-mesh sieve, were accurately weighed and precisely mixed for 10 minutes using a mortar. This blending resulted in a homogeneous physical mixture (PM). Subsequently, the obtained mixtures were stored within a desiccator at room temperature for further evaluation [9].

**2.1.1 Solid dispersion.** Solid dispersions of glipizide in combination with the polymers (PVP K30, K90, and PEG 6000) were formulated, employing various weight ratios of 1:1, 1:2, 1:3, and 1:4, through the solvent evaporation technique (Table 1). The predetermined quantity of the selected polymer was dissolved in dichloromethane (DCM) at ambient temperature, with a continuous and vigorous stirring at 500 rpm utilizing a magnetic stirrer (IKA Werke, RT 10 Power, Germany). Once a clear solution was achieved, the designated amount of the glipizide drug was gradually introduced in portions, and the ensuing mixture was subject to uninterrupted stirring for a duration of one hour to ensure the formation of a transparent solution. Subsequently, the solvent was carefully evaporated under temperature of 35°C. The resulting co-precipitate was meticulously collected from the beaker, subjected to grinding in a mortar, sieved through a 355 μm mesh, and stored in a desiccator containing CaCl_2_ for preservation [9, 16, 17–23].

**Table 1:** Composition of glipizide solid dispersions and physical mixtures.

Formulation	Polymer	Drug-Polymer Ratio	Drug (mg) / Polymer (mg)
PM F	PVP K30	1:2	300/600
PM FP	PVP K90	1:2	300/600
PM G	PEG 6000	1:2	300/600
F1	PVP K30	1:1	300/300
F2	PVP K30	1:2	300/600
F3	PVP K30	1:3	300/900
F4	PVP K30	1:4	300/1200
FP1	PVP K90	1:1	300/300
FP2	PVP K90	1:2	300/600
FP3	PVP K90	1:3	300/900
FP4	PVP K90	1:4	300/1200
G1	PEG 6000	1:1	300/300
G2	PEG 6000	1:2	300/600
G3	PEG 6000	1:3	300/900
G4	PEG 6000	1:4	300/1200

#### 2.2 Determination of saturation solubility

Saturation solubility investigations were conducted on both pure glipizide and samples of solid dispersion to quantify the percentage enhancement in glipizide’s solubility. Excess amounts of the samples (glipizide solid dispersions and pure glipizide) were introduced into 25 ml volumetric flasks containing 10 ml of pH 6.8 phosphate buffer. The mixtures were agitated at 100 rpm on a magnetic stirrer for a 24-hour period at room temperature. Following this, the resulting suspensions underwent filtration through a 0.45 μm filter. Utilizing spectrophotometric analysis at 275 nm, the concentration of glipizide was determined. This solubility assessment was performed in triplicate for each sample [12, 15]. The optimization of polymer-to-drug ratios was guided by achieving the maximum solubility and dissolution rate [24].

#### 2.3 Preparation of tablets by direct compression

Tablets of glipizide and two optimized formulations (F4 and FP4) with highest solubility were formulated by direct compression method using a single-punch tablet machine (Emmy, Pakistan). Glipizide and optimized formulations of solid dispersion (F4 and FP4) was triturated with 1% talc, 0.5% magnesium stearate and lactose (as filler) for 15 to 20 minutes. Each mixture was compressed separately into tablets (GP, TF4 and TFP4, respectively) by direct compression on a single punch tablet machine [25].

#### 2.4 Evaluation of tablets

**2.4.1 Weight variation and content uniformity.** Uniformity of weight was determined by evaluating 20 tablets separately and their average was calculated. Subsequently, the weight of each individual tablet was contrasted with the average weight. Tablets adhere to the USP guidelines if the number of tablets exceeding the specified percentage limit is limited to a maximum of two. Additionally, no tablet should exhibit a deviation greater than twice the prescribed percentage limit [11].

To determine the uniformity of content, ten tablets from each formulation were individually weighed, and then placed each tablet in a separate 1000 mL volumetric flask. These flasks contained a mixture of methanol and water in a 1:1 ratio. After the sonication process, the solutions of samples were filtered through 0.45 μm filters and analyzed using a UV spectrophotometer (Shimadzu, Japan) set at a wavelength of 275 nm. This procedure was conducted in triplicate, and the resulting data was presented as the mean ± standard deviation [18].

**2.4.2 Friability.** The friabilator (manufactured by Roche, Germany), was used to evaluate the combined impact of abrasion and shock. This is achieved through a plastic chamber that rotates at a speed of 25 revolutions per minute (rpm), causing the tablets to drop from a distance of six inches during each rotation. Tablets equivalent to 6.5 mg weight were taken and placed into the apparatus, then apparatus was run for 100 revolutions. Then tablets were dusted and their weight was determined, 0.5–0.1% loss in weight is generally acceptable [11].

**2.4.3 Hardness.** Tablets must possess a certain degree of strength or hardness to withstand the mechanical impact they experience throughout the processes of production, packaging, and transit. The hardness of 20 tablets from each batch was determined by using monsanto hardness tester (Emmay, Pakistan) then mean and relative standard deviation was determined [9].

**2.4.4 Thickness and diameter.** Thicknesses and diameter of 10 tablets were measured by using Vernier Caliper (Emmay, Pakistan).

**2.4.5 Disintegration test.** The disintegration time was determined using a tablet disintegration tester equipped with a Basket-rack assembly. A single unit of the dosage was positioned within each of the six tubes of the basket. The disintegration process was carried out in phosphate buffer with a pH of 6.8, serving as the disintegration medium, and maintained at a temperature of 37 ± 0.5°C [26].

#### 2.5 In vitro dissolution of solid dispersions

Dissolution investigations for the solid dispersion were conducted using a USP type II (paddle) apparatus, operating at a rotation speed of 100 rpm. Samples of solid dispersion, containing an equivalent of 5 mg of glipizide, were examined in 900 mL of phosphate buffer at a pH of 6.8, and the temperature was controlled at 37 ± 0.5°C. The dissolution experiment lasted for one hour, during which samples were collected at appropriate time intervals: 5, 10, 15, 30, 45, and 60 minutes. After each sampling, an equivalent volume of dissolution medium was replenished. These samples were subjected to analysis using a single beam spectrophotometer set at 275 nm to quantify glipizide concentration, which was then compared against a calibration curve. This allowed the construction of profiles illustrating the percentage of drug released over time [9]. Different kinetic models were applied to dissolution data to study the drug release kinetics.

#### 2.6 In-vitro dissolution of tablets

Dissolution studies were conducted utilizing a USP paddle type-II apparatus (DBK Instruments, Mumbai). The experiments were carried out in a medium of 6.8 pH phosphate buffer, comprising 900 ml. The conditions included a rotation speed of 100 rpm and a controlled temperature of 37 ± 0.5°C. During the study, tablets were immersed in the dissolution medium, and at specific time points (5, 10, 15, 30, 45, and 60 minutes), 5 ml samples were removed. These samples were subsequently subjected to analysis using a single beam spectrophotometer set to a wavelength of 275 nm [27]. Different kinetic models were applied to dissolution data to study the drug release kinetics.

#### 2.7 Characterization of solid dispersion

**2.7.1 Fourier transformed infrared spectroscopy (FTIR).** FTIR spectroscopic analysis was done on the solid dispersions to check potential interactions between the drug and the polymers. In order to analyze the samples in a solid state, their FTIR spectra were obtained using the potassium bromide (KBr) disc technique. The Fourier transform infrared spectroscopy (FTIR) spectra of glipizide, PVP K30, PVP K90, and the optimized solid dispersions (F4 and FP4) were captured using an FTIR-840S (Shimadzu, Japan). The samples were prepared by embedding them in KBr disks (with 2 mg of sample and 200 mg of KBr) and then placed in a sample holder for examination. Scanning was conducted across the range of 500-4000/cm, employing a resolution of 2/cm and a scan speed of 64 scans per second. The data was recorded using the IRSolution software (Shimadzu, Japan) [28].

**2.7.2 X-ray diffraction.** X-ray diffraction is a non-destructive technique [12]. Currently, it holds a significant position as an analytical instrument within the pharmaceutical industry, offering insights of both qualitative and quantitative nature into the molecular structure. The technique of X-ray diffraction plays a crucial role in attaining comprehensive knowledge of the physical, chemical, and structural attributes inherent to diverse materials such as polymers, metals, and other solid substances. To delve into polymorphic changes triggered by solid dispersion formulation, the powder X-ray diffractograms (PXRDs) of glipizide, PVP K30, PVP K90, as well as the optimized solid dispersions (F4 and FP4), were meticulously recorded. This insightful analysis was executed employing an X-ray diffractometer, JDX-3532 (JEOL, Japan) [29].

**2.7.3 Differential scanning calorimetry.** Differential scanning calorimetry (DSC) thermograms for glipizide, PVP K30, PVP K90, and the optimized solid dispersions (F4 and FP4) were obtained using a DSC 404 C Pegasus instrument (NETZSCH, USA). The instrument was calibrated using indium. The samples underwent examination with a heating rate of 10°C per minute, spanning the temperature range of 10°C to 300°C, all within a nitrogen atmosphere (with a flow rate of 20 mL per minute) [30].

## 3. Results and discussion

### 3.1 Saturation solubility

Saturation solubility was determined to check the improvement in glipizide solubility. It was observed that each polymer used (i.e. PVP K30, PVP K90, and PEG 6000) had increased the solubility of glipizide (Table 2). Solid dispersion (F4) prepared from PVP K30 showed 95.13% increase in solubility. PVP K90 also increased the solubility to 92.60%. Solid dispersions prepared by PEG 6000 improved the solubility to 89.12%. These results revealed that solid dispersions prepared by PVP K30 showed more solubility enhancement than PVP K90 and PEG 6000.

**Table 2:** Saturation solubility of formulations.

Formulation	Solubility in Phosphate buffer pH 6.8 (μg/ml)	Increase in solubility (%)
**Glipizide**	18.49	-
**F1**	200	90.76
**F2**	230	91.96
**F3**	270	93.15
**F4**	380	95.13
**FP1**	98	81.1
**FP2**	145	86.97
**FP3**	217	91.48
**FP4**	250	92.60
**G1**	70	73.59
**G2**	90	79
**G3**	130	85.78
**G4**	170	89.12

PVP K30 showed more solubility enhancement than PVP K90, this may be because of high molecular weight and high viscosity of PVP K90 [12]. In a previous study on allopurinol same results have been reported [31]. It was also observed that PVP improved solubility more efficiently than PEG. In another study PVP K30, PVP K90, and PEG 6000 were compared and PVP K30 showed more solubility enhancement [32]. Narang also reported that PVP enhance solubility more effectively than PEG [33].

#### 3.2 Evaluation of tablets

The physical attributes of the tablets, encompassing weight variation, visual appearance, tablet hardness, tablet thickness, friability, and disintegration time of the formulated solid dispersions, were found to be satisfactory, as outlined in Table 3. Tablet hardness values ranged between 6.99 and 7.09, demonstrating consistent tablet firmness. The friability percentage registered at less than 0.5%, indicating resilience against transportation-related stress. Furthermore, the disintegration times for all batches remained under 15 minutes, in accordance with the stipulated USP limits. Each batch exhibited weight variation, thickness, and diameter within the prescribed range of ± 5%, adhering to the requirements set by USP. All tests conducted successfully met the stipulated criteria as per USP guidelines.

**Table 3:** Evaluation of physical properties of tablets.

Formulation	Weight Variation (mg), n = 20 Mean ± S.D	Content Uniformity (mg), n = 10 Mean ± S.D	Thickness (mm) n = 10, Mean ± S.D	Diameter (mm) n = 10 Mean ± S.D	Hardness (Kg/cm^2^) n = 10 Mean ± S.D	Friability (%) n = 16 Mean ±S.D	Disintegration Time (min) Mean ±S.D
**Glipizide**	400.9 ± 1.7	9.8 ± 0.9	3.01 ± 0.114	10.4 ±0.126	7.02 ± 1.12	0.4 ± 0.02	5 ± 0.03
**F4 tablet**	402.15 ±1.4	9.3 ±1.1	3.06 ± 0.117	10.2 ±0.121	7.09 ± 1.18	0.3 ± 0.06	7 ± 0.06
**FP4 tablet**	400.08 ±1.9	9.5 ±1.3	3.11 ± 0.113	10.6 ±0.127	6.99 ± 1.14	0.4 ± 0.06	7 ± 0.04

#### 3.3 In-vitro dissolution of solid dispersion

**Fig 1:**
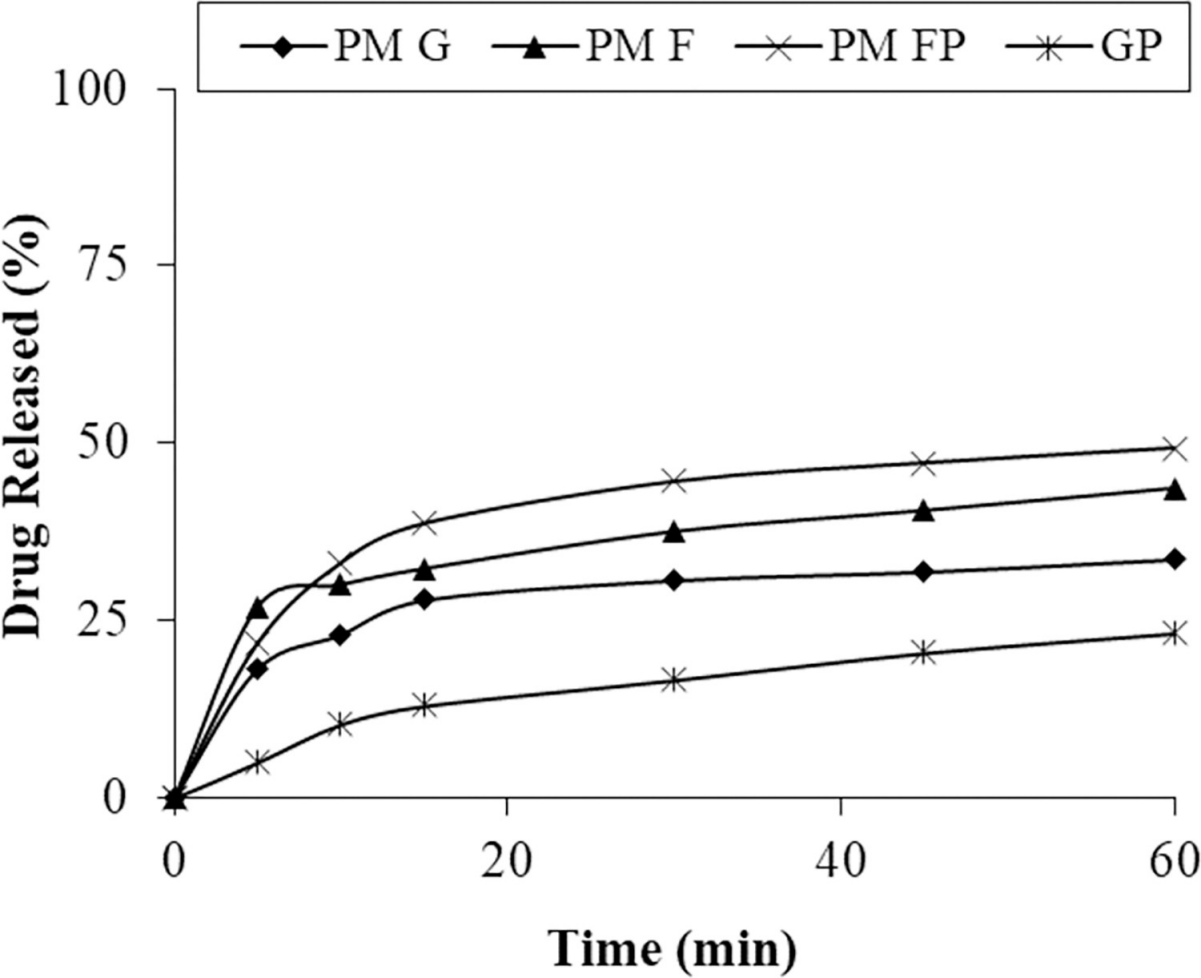
Dissolution profile of pure drug and PMs of each polymer.

**Fig 2:**
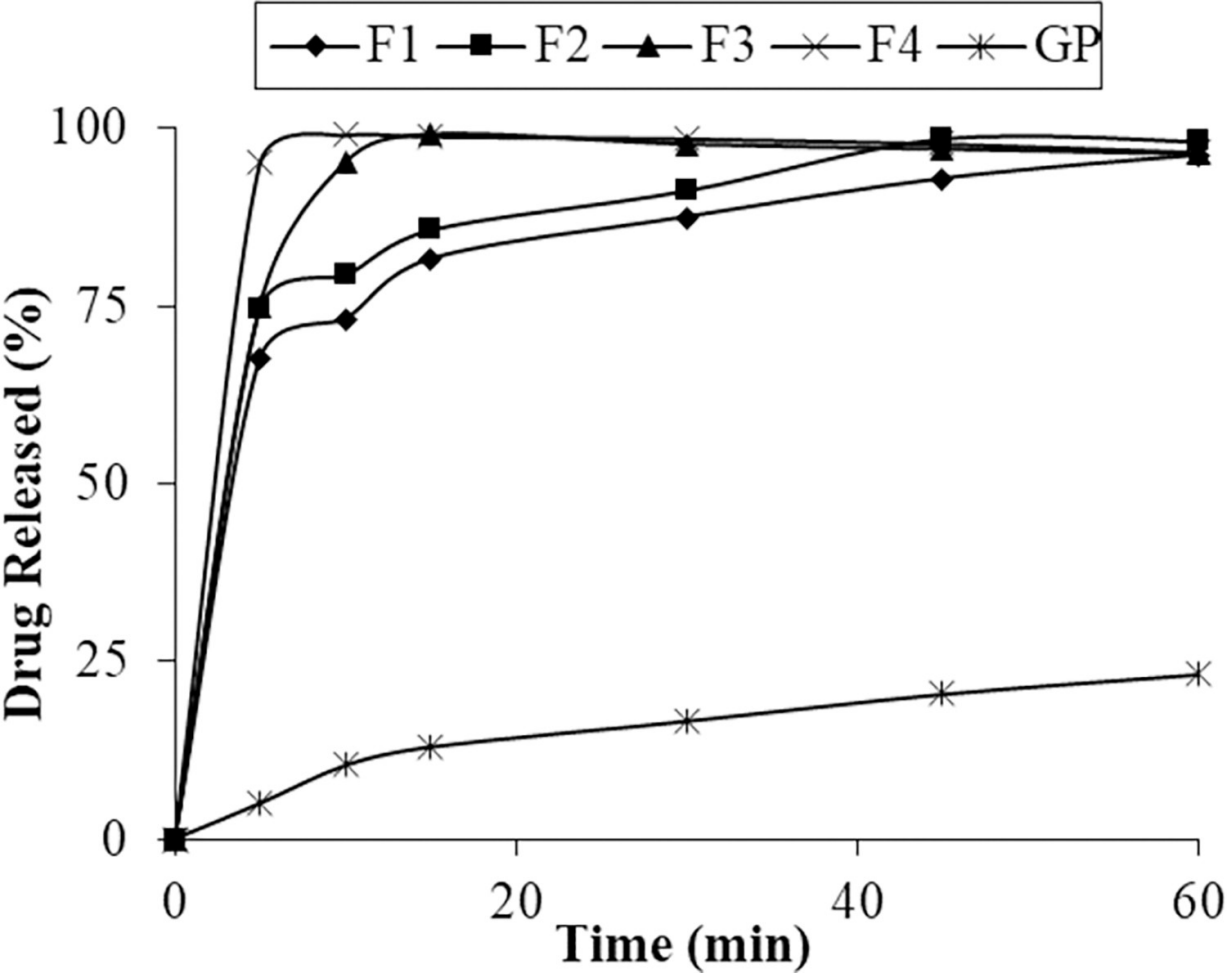
Dissolution profile of pure glipizide and SD of PVP K30.

*In-vitro* drug dissolution studies were done to determine the dissolution profile of all the prepared formulations. The in-vitro dissolution was performed in phosphate buffers of pH 6.8 at temperature 37.0 ± 0.5°C continued for 60 minutes. Drug dissolution from PVP K30 solid dispersion was higher and rapid (Fig 1). The drug dissolved after 30 minutes from formulations F1, F2, F3, and F4 were 87.47%, 91.32%, 97.74% and 98.58% respectively at pH 6.8. While drug dissolution of respective physical mixture was less than 40% at pH 6.8 after 30 minutes (Figs 1 and 2).

F4 (1:4) showed rapid and increased dissolution than F1 (1:1), this revealed that dissolution is directly proportional to the concentration of polymer i.e. PVP K30. In a similar study Meka et al. found that higher polymer concentration results in higher percent drug release [24]. In a separate investigation, it was noted that raising the concentration of PVP in the dispersion leads to a corresponding rise in the release rate [34]. Kearney et al. reported similar behavior [35]. Ning et al. also reported that increase in concentration of PVP K30 results in increased dissolution and solubility [36]. Additionally, another research study indicated that as the concentration of the carrier was further elevated, the rate of release exhibited a deceleration. Notably, the release rate displayed an increase at a drug/carrier ratio of 1:4, but this trend reversed at ratios of 1:5 and 1:6. In this case, the 1:4 ratio proved to be optimal for the formulation in PVP K30 [14].

**Fig 3:**
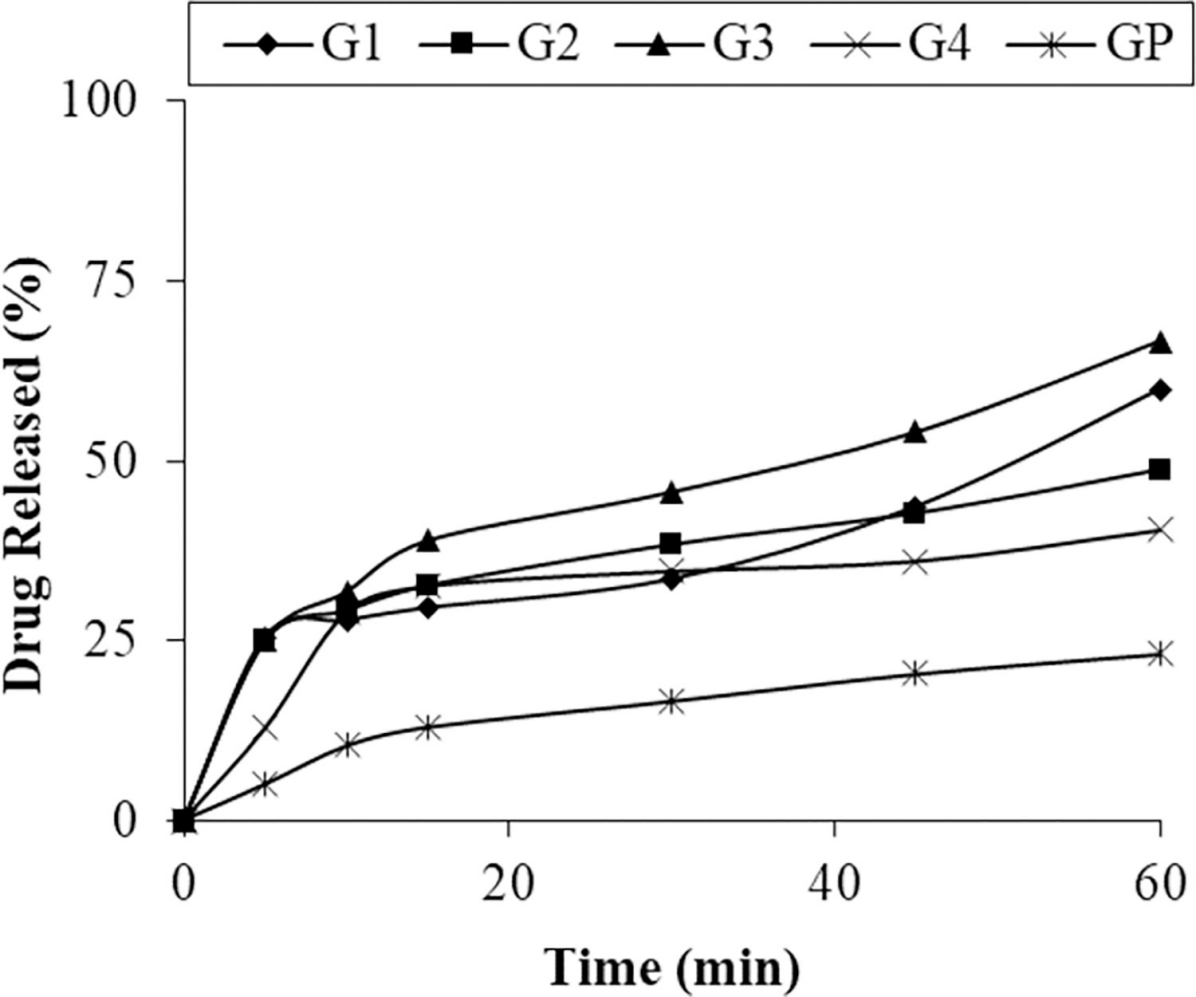
Dissolution profile of pure glipizide and SD of PEG 6000.

The *in-vitro* dissolution pattern of PEG 6000 formulations (G1, G2, G3, G4) were showing less dissolution and sustained release pattern. After 60 minutes the amount of glipizide dissolved in medium were < 70% in phosphate buffer pH 6.8 (Fig 3). It was observed that formulation G3 showing increased dissolution i.e. 66.66%. Physical mixture of PEG 6000 and drug (1:2) showed 33.67% dissolution after 1 hour (Fig 1). It was observed that PEG 6000 increased the dissolution but showed sustained release effect. Isaac et al. reported the same results in a previous study [29]. In a similar study Narang and Sirivastava reported that PEG 6000 showed less dissolution than other grade e.g. PEG 4000 and PEG 9000. In the same study they reported more solubility and dissolution of drug is increased by PVP as compared to PEG [24, 28].

**Fig 4:**
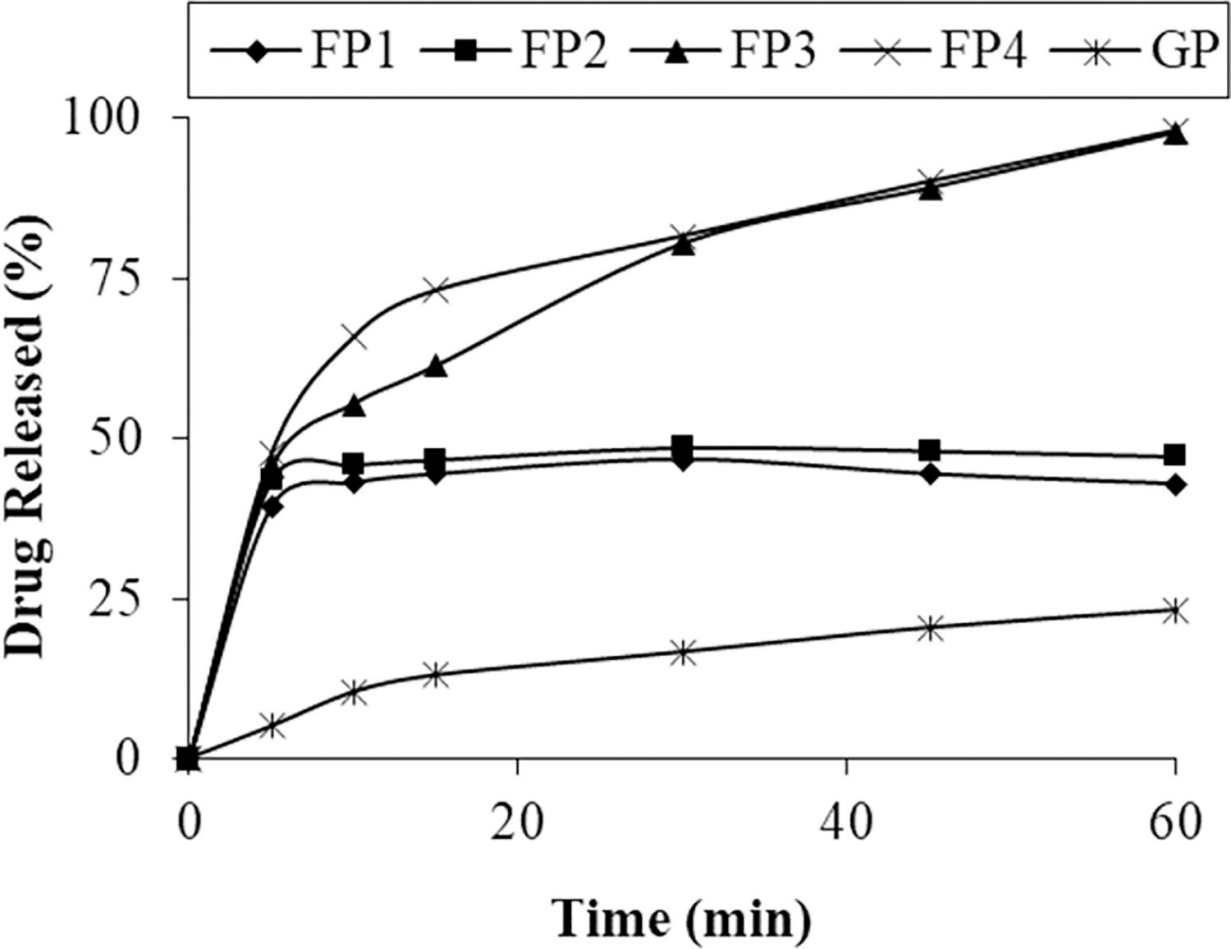
Dissolution profile of pure glipizide and SD of PVP K90.

Drug dissolution from PVP K90 solid dispersions were higher but slower than PVP K30 (Fig 4). The drug dissolved after 30 minutes form formulations FP1, FP2, FP3, and FP4 were 46.69%, 48.61%, 80.38% and 81.57% respectively at pH 6.8. While drug dissolution of respective physical mixture was 44.60% at pH 6.8 after 30 minutes (Fig 1).

Dissolution profile showed that increase in concentration of polymer results in increase in dissolution. Mehramizi et al. reported similar behavior of PVP K90 solid dispersion prepared by spray drying method [28]. PVP K90 showed increased dissolution but slow drug release. It was noted that PVP K90 showed slow drug release as compared to PVP K30. Similar behavior was reported in a previous study [12].

The dissolution profile reveals that even the physical mixtures contributed to enhanced dissolution, possibly attributed to an improved drug wettability and the polymer carrier’s role in curbing drug particle aggregation [36]. But solid dispersions improved dissolution and solubility as compared to physical mixtures [9, 19].

The solid dispersion formulations F4 and FP4 were selected on the basis of percent increase in solubility and dissolution profile. Solid dispersion F4 enhanced the solubility of glipizide to 95.13% and showed 99.10% drug release in 10 minutes of dissolution. Solid dispersion FP4 increased the solubility up to 92.60% while 98.06% drug was dissolved in 60 minutes. Selected formulations were further characterized and converted into solid oral dosage form.

#### 3.4 In-vitro dissolution of tablets

**Fig 5:**
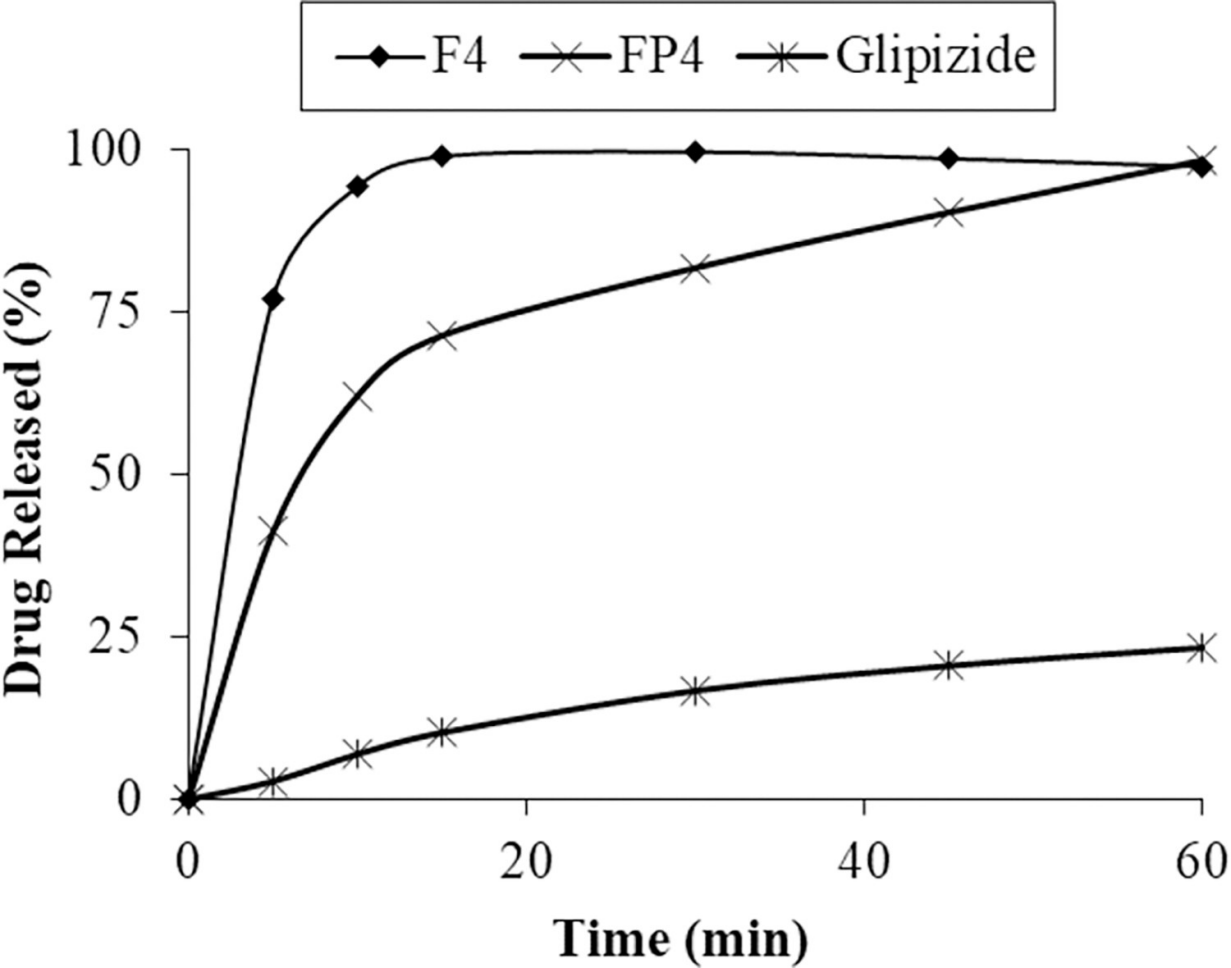
Dissolution profile of tablets.

The dissolution test provided valuable insights into the dissolution behaviors exhibited by the formulated tablet variants. Among them, the tablet comprising a solid dispersion of glipizide and PVP K30 (F4, 1:4) demonstrated the swiftest dissolution rate, followed closely by the tablet containing the solid dispersion of glipizide and PVP K90 (FP4, 1:4). In contrast, tablets containing pure glipizide exhibited a notably sluggish dissolution process along with limited solubility, as visually represented in Fig 5.

Tablets containing optimized solid dispersions, engineered to possess enhanced solubility, showcased favorable dissolution profiles, with up to 80% of the drug being released into the dissolution medium within a span of 30 minutes. This achievement successfully met the acceptance criteria for immediate-release tablet dissolution, thereby satisfying the specified threshold of 75% (Q min).

#### 3.5 Kinetic modeling of in-vitro drug release

For ascertaining the sequence and mechanism governing drug release, the in-vitro drug release dataset was subjected to fitting within diverse kinetic models, encompassing Zero and First order, Higuchi, and Korsmeyer-Peppas models. As elucidated by the data presented in Table 4, the First order values surpassed those of the Zero order, thereby establishing the concentration-dependency of in-vitro drug release. Likewise, across all formulations, the Higuchi model values exceeded the threshold of 0.45, thereby indicating the prevalence of a diffusion-driven phenomenon. In case of Korsmeyer-Peppas model, all the “n” values were above 0.45 which proved that drug release followed non-Fickian behavior.

**Table 4:** Kinetic modeling of in-vitro drug release data.

Formulation	Code	R^2^				Value of “n” for Korsmeyer-Peppas
		Zero-Order	First-Order	Higuchi Model	Korsmeyer-Peppas Model	
F	1	0.4946	0.9007	0.7631	0.6997	0.9826
	2	0.4464	0.8909	0.7192	0.6865	0.9855
	3	0.3057	0.2772	0.5871	0.6661	0.9836
	4	0.215	0.1208	0.4722	0.6307	0.966
FP	1	0.2535	0.2628	0.5278	0.6501	0.8077
	2	0.2697	0.2895	0.5404	0.6492	0.8222
	3	0.7687	0.9603	0.955	0.7811	1.0078
	4	0.667	0.9416	0.8964	0.7575	1.0051
G	1	0.8299	0.883	0.9207	0.7984	0.8632
	2	0.7175	0.8115	0.9217	0.7794	0.8465
	3	0.8443	0.9345	0.9765	0.8257	0.9222
	4	0.6323	0.6833	0.8522	0.8291	0.8509
TABLETS	F4	0.3156	0.3636	0.5985	0.6669	0.9861
	FP4	0.7136	0.9536	0.9253	0.7826	1.0177
	GP	0.9459	0.9596	0.9722	0.984	0.8081

#### 3.6 Characterization of solid dispersion

**Fig 6:**
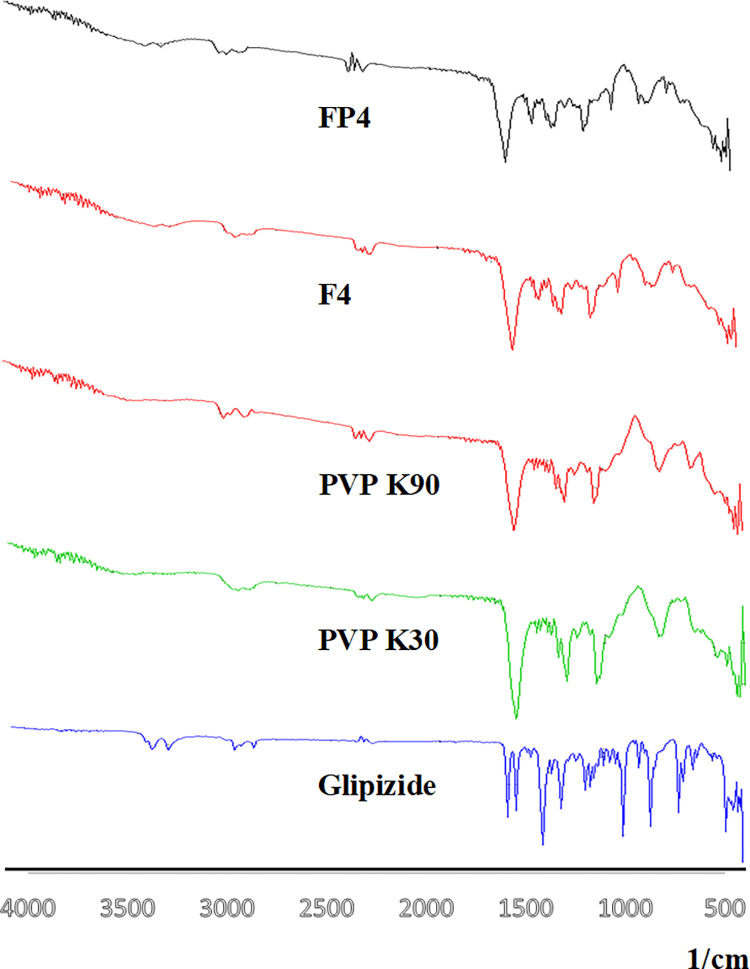
FTIR spectrum of glipizide, PVP K30, PVP K90 and optimized solid dispersions.

**3.6.1 FTIR.** Fig 6 illustrates the FTIR spectra of both glipizide and the individual polymers, along with their corresponding solid dispersions. In the case of glipizide, distinctive peaks were observed at 1649 cm^-1^ for ureaide (O = C-NH_2_) and 1688 cm^-1^ for the amid group. Additional discernible bands were present at 1159 cm^-1^ and 1527 cm^-1^ (C = C), signifying the stretching of the S = O bond within the SO_2_ moiety. Yet, specific peaks were absent in the spectra of PVP K30 and PVP K90, a result of their substantial molecular weight. On the other hand, the absorption bands that correlated with the drug substantiated its crystalline attributes.

Upon examining the FTIR spectra of the solid dispersions, modifications in the peak associated with carbonyl functionality were observed. This change could likely be attributed to hydrogen bonding effects. Notably, this observation aligns with findings reported by other researchers [29, 30].

**Fig 7:**
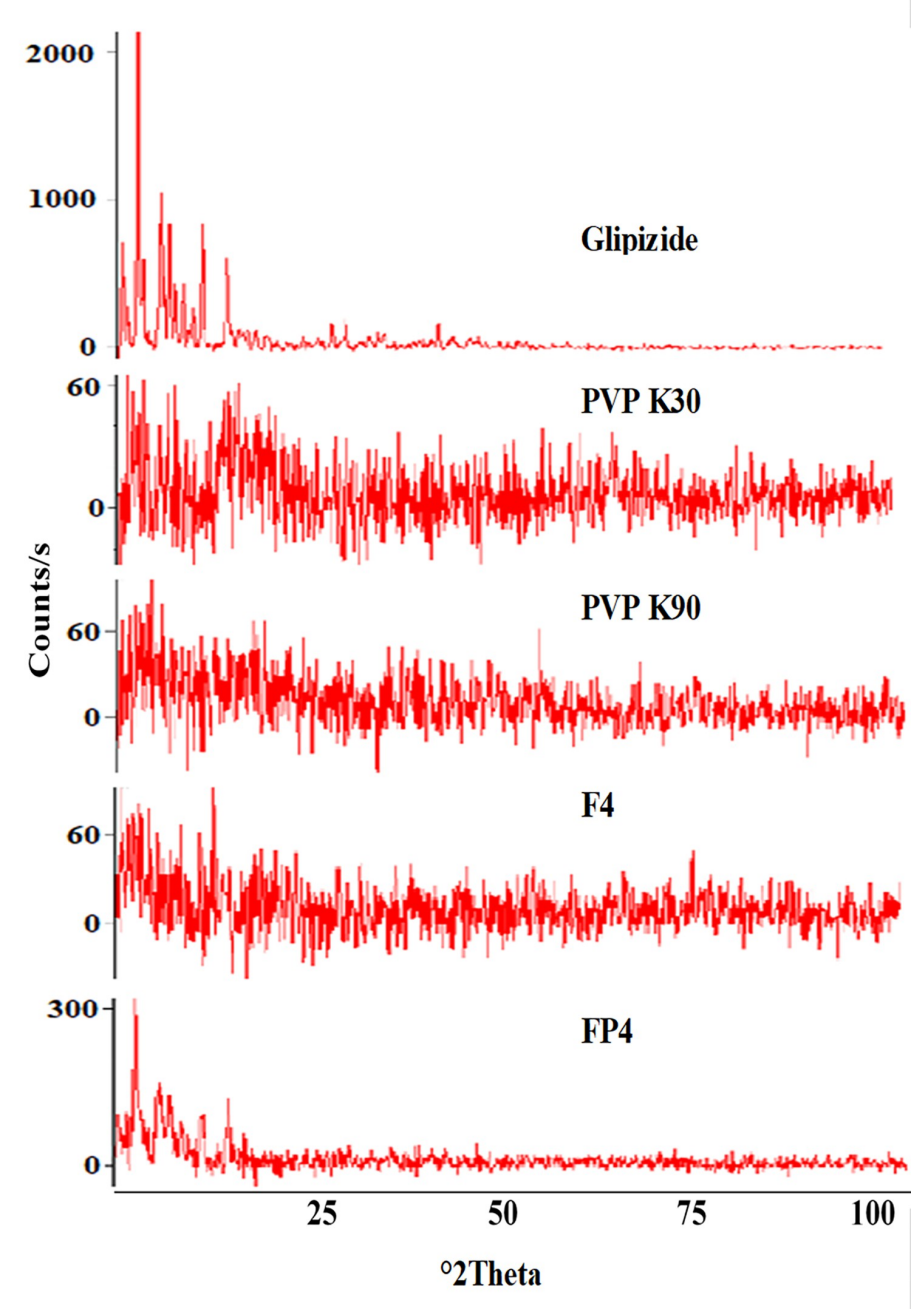
XRD diffractogram of glipizide, PVP K30, PVP K90 and optimized solid dispersions.

**3.6.2 X ray diffraction.** The XRD patterns for the glipizide, polymers, and the optimized solid dispersions are shown in Fig 7. X-ray diffractogram of pure glipizide showed sharp peaks which confirmed the crystalline nature of drug. The absence of diffraction peaks of glipizide in optimized solid dispersion’s diffractogram proposed the development of drug-polymer complex in the solid state and conversion of crystalline structure into amorphous form. Mehramizi et al. reported similar results [28].

**Fig 8:**
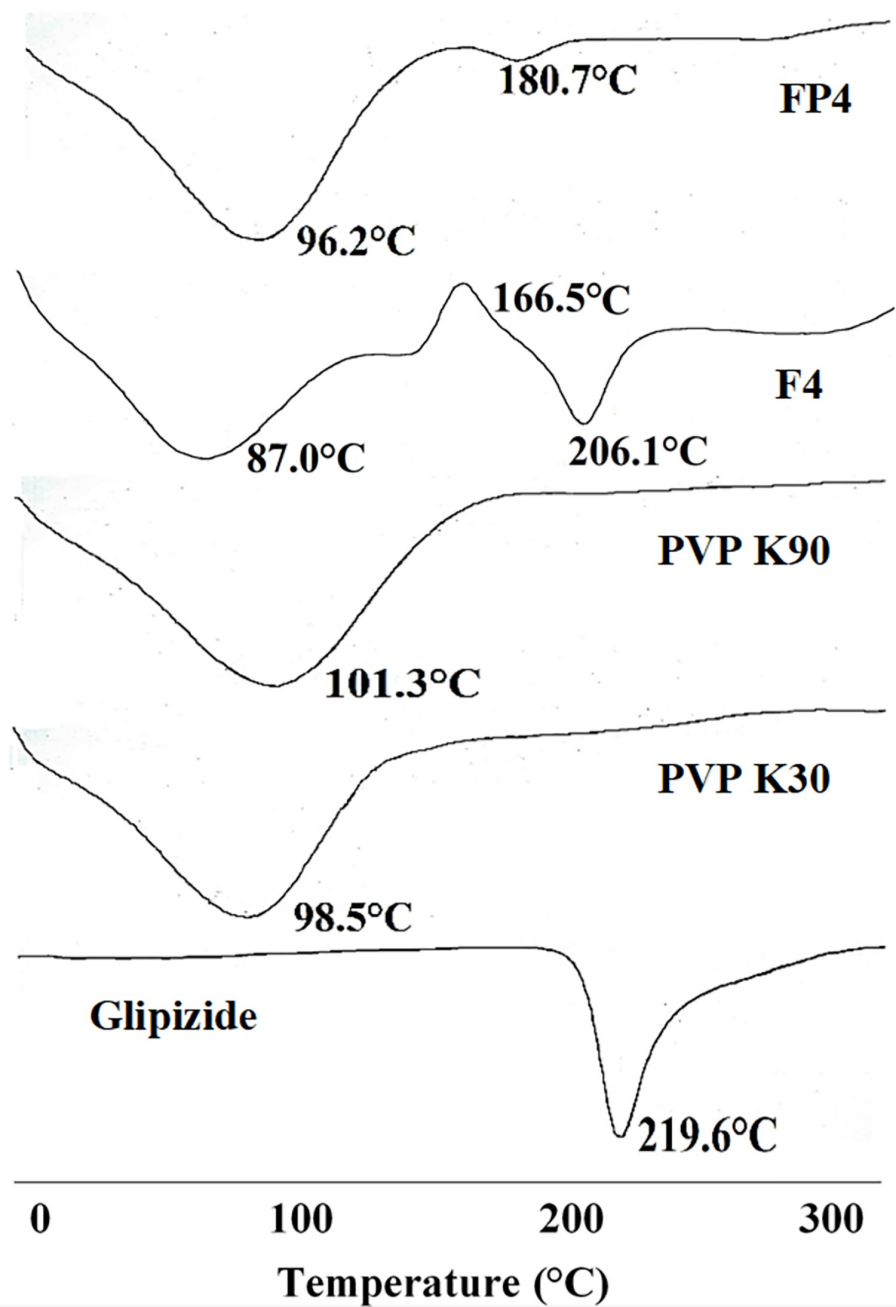
DSC of glipizide, PVP K30, PVP K90 and optimized solid dispersions.

**3.6.3 Differential scanning calorimetry.** Fig 8 presents the DSC thermograms obtained for glipizide, PVP K30, PVP K90, and the solid dispersions (SDs) prepared using PVP. When examining the thermogram of powdered glipizide, a distinct endothermic peak indicative of melting was observed at a temperature of 219.6°C. In contrast, PVP K30 and PVP K90 exhibited their own separate endothermic peaks at lower temperatures of 98.5°C and 101.3°C, respectively.

In the thermograms of formulations F4 and FP4, the intensity of the endothermic peaks associated with PVP K30 and PVP K90 was notably more pronounced. Notably, the DSC curve of the F4 solid dispersion displayed the presence of the glipizide melting peak, albeit with a discernible shift of 13.5°C. This shift suggests a reduction in the crystalline nature of the drug, potentially stemming from interactions occurring between the different components. This finding aligns with results reported by researchers Sethia and Squillante [37, 38]. Within the thermogram of the F4 formulation, an additional peak becomes evident around 160°C. This observation suggests that a minor quantity of the drug has permeated the polymer phase, leading to the formation of less well-structured crystals with a subsequently lower melting point. This phenomenon was observed in a study conducted by Alves et al., where similar findings were reported [39, 40]. Contrastingly, within the endothermic curve of FP4, the intensity of the glipizide’s endothermic peak was scarcely discernible, occurring around 180.7°C. A recognized phenomenon arises when PVP is employed in solid dispersions, wherein its comparatively lower melting point facilitates the dissolution of drug particles into the polymer melt. This process can lead to the formation of monotectic systems prior to the drug’s actual melting point being attained. Similar findings were documented by Mehramizi et al. and Tantishaiyakul et al., further validating this observation [9, 19]. Upon analyzing the melting points of polymers within solid dispersions, it becomes apparent that the melting point of PVP K30 and PVP K90 undergoes a downward shift. This phenomenon arises as a result of the formation of a binary system encompassing the drug and the matrix [30, 41, 42].

## 4. Conclusion

The process of solvent evaporation was employed to fabricate solid dispersions of glipizide utilizing three distinct polymers: PEG 600, PVP K30, and PVP K90. This method yielded an enhanced product, displaying notable advancements in both the drug’s solubility and dissolution characteristics. The outcomes of this study underscore a remarkable enhancement in the dissolution rate of glipizide, which transitioned from nearly negligible levels for the pure drug to an impressive 90%. Moreover, all formulated solid dispersions performed better than their corresponding physical mixtures in terms of solubility and dissolution. Using techniques such as FTIR spectroscopy, X-ray diffraction (XRD), and differential scanning calorimetry (DSC), the study revealed compelling evidence of a physical interaction between the drug and polymer, while simultaneously ruling out any chemical degradation of the drug.
